# Intra-image referencing for simplified assessment of HER2-expression in breast cancer metastases using the Affibody molecule ABY-025 with PET and SPECT

**DOI:** 10.1007/s00259-017-3650-3

**Published:** 2017-03-06

**Authors:** Dan Sandberg, Vladimir Tolmachev, Irina Velikyan, Helena Olofsson, Anders Wennborg, Joachim Feldwisch, Jörgen Carlsson, Henrik Lindman, Jens Sörensen

**Affiliations:** 1 Department of Immunology, Genetics and Pathology, Uppsala University, Uppsala, Sweden; 2 Section of Nuclear Medicine and PET, Department of Surgical Sciences, Uppsala University, Uppsala, Sweden; 3 Affibody AB, Solna, Sweden

**Keywords:** Affibody, HER2-receptor, Shedding, PET, SPECT, T/R

## Abstract

**Purpose:**

In phase I/II-studies radiolabelled ABY-025 Affibody molecules identified human epidermal growth factor receptor 2 (HER2) expression in breast cancer metastases using PET and SPECT imaging. Here, we wanted to investigate the utility of a simple intra-image normalization using tumour-to-reference tissue-ratio (T/R) as a HER2 status discrimination strategy to overcome potential issues related to cross-calibration of scanning devices.

**Methods:**

Twenty-three women with pre-diagnosed HER2-positive/negative metastasized breast cancer were scanned with [^111^In]-ABY-025 SPECT/CT (n = 7) or [^68^Ga]-ABY-025 PET/CT (n = 16). Uptake was measured in all metastases and in normal spleen, lung, liver, muscle, and blood pool. Normal tissue uptake variation and T/R-ratios were established for various time points and for two different doses of injected peptide from a total of 94 whole-body image acquisitions. Immunohistochemistry (IHC) was used to verify HER2 expression in 28 biopsied metastases. T/R-ratios were compared to IHC findings to establish the best reference tissue for each modality and each imaging time-point. The impact of shed HER2 in serum was investigated.

**Results:**

Spleen was the best reference tissue across modalities, followed by blood pool and lung. Spleen-T/R was highly correlated to PET SUV in metastases after 2 h (r = 0.96, P < 0.001) and reached an accuracy of 100% for discriminating IHC HER2-positive and negative metastases at 4 h (PET) and 24 h (SPECT) after injection. In a single case, shed HER2 resulted in intense tracer retention in blood. In the remaining patients shed HER2 was elevated, but without significant impact on ABY-025 biodistribution.

**Conclusion:**

T/R-ratios using spleen as reference tissue accurately quantify HER2 expression with radiolabelled ABY-025 imaging in breast cancer metastases with SPECT and PET. Tracer binding to shed HER2 in serum might affect quantification in the extreme case.

**Electronic supplementary material:**

The online version of this article (doi:10.1007/s00259-017-3650-3) contains supplementary material, which is available to authorized users.

## Introduction

Breast cancer is a disease of major socio-economic importance with a lifetime risk of 12% [1]. Strategic therapy decisions and prognosis can be improved by subdividing the disease histologically. Immunohistochemistry (IHC) is used to evaluate the expression of hormone receptors such as oestrogen (ER), progesteron (PR), and the transmembrane receptors human epidermal growth factor 2 (HER2) [2]. Contrary to the first two, HER2 has no known natural ligand and signals by hetero-dimerization with activated members of HER family receptors [3]. Overexpression of HER2 has an incidence of 15-20% in breast cancer [4–6] and is associated with poorer prognosis [4, 6]. Therapy using monoclonal antibodies such as trastuzumab, pertuzumab, and more recently the antibody-drug conjugate trastuzumab-emtansine improves progression-free survival and overall survival of patients with HER2-positive breast and gastroesophageal cancer [4, 6, 7].

Diagnosing HER2 receptor expression is crucial to identify patients expected to benefit from treatment, as well as to avoid unnecessary cost and potential risk of serious adverse effects of treatment for patients with HER2-negative tumours. Growing evidence of heterogeneous HER2 expression complicates the clinical decision making, as immunohistochemistry (IHC) or in situ hybridization (ISH) analysis of the primary tumour is unreliable as sole source of information in the metastatic setting [5, 8–11]. Current guidelines recommend biopsy with renewed IHC and/or ISH evaluation from at least one metastasis before decision about further HER2-targeted therapy [12]. Metastases may, however, not be easily available for biopsy procedures. Biopsy sampling from metastases is, even in the ideal case, associated with discomfort for the patient, potential risk for infection and hemorrhage, and is for such reasons sometimes omitted. Biopsy is generally guided by morphological imaging methods like CT or ultrasound and may miss the viable part of a tumour mass resulting in non-representative and misleading information.

Radionuclide molecular imaging-based methods offer non-invasive, whole body based diagnostics with minimal patient discomfort while also being repeatable for monitoring of the disease and therapy [13–15]. The use of radiolabelled therapeutic anti-HER2 antibodies as imaging agents is an obvious possibility. However, intact antibodies have a slow blood clearance and slow tumour penetration resulting in an optimal imaging time point of 4-5 days after injection [14]. Furthermore, macromolecules accumulate in tumours due to enhanced vascular permeability, irrespective of molecular target expression [16], potentially causing false-positive findings. An alternative approach is to use radiolabelled HER2-specific Affibody^®^ molecules as imaging agents. Affibody molecules are engineered scaffold peptides, which can be selected for high affinity binding to a variety of proteinaceous molecular targets [17]. The small molecular size (6-7 kDa peptide) is fundamental for fast kinetics with rapid clearance from both blood and tumours with low HER2-expression, which is needed for high contrast imaging with limited radiation dose using short-lived isotopes.

To be reliable, a HER2 imaging method should address two issues, regardless of tracer choice. Firstly, only tumours with HER2 expression matching 3+ staining by IHC or 2+ and ISH-positive are considered as HER2-positive and would respond to anti-HER2 therapy [2]. Tumours with lower expression levels would not respond to the targeted therapy, but they may contain tens or even hundreds of thousands HER2 receptors per cancer cell [18] and might be detected by radionuclide imaging [19]. Thus, the imaging method should reliably reflect the receptor concentration to allow correct classification of a HER2-negative tumour despite it also expressing the receptor to some extent. Secondly, the shed form of the HER2 receptor is present in the blood circulation with normal levels of serum-HER2 lower than 15 μg/L. On occasion much higher values can occur, primarily due to shedding from necrotic tumour cells. In such cases, any HER2-specific molecule introduced in the blood circulation might to some extent be trapped there with the potential of altering its expected tissue distribution.

ABY-025 is a second generation HER2-binding Affibody molecule with improved scaffold and reduced non-specific liver uptake [20–22]. With high affinity for domain 3 of the extracellular portion of the HER2 receptor it has the important advantage of not competing for binding with trastuzumab (domain 4) and pertuzumab (domain 2), permitting diagnostic imaging during HER2-directed antibody therapy [23, 24].

**Fig. 1:**
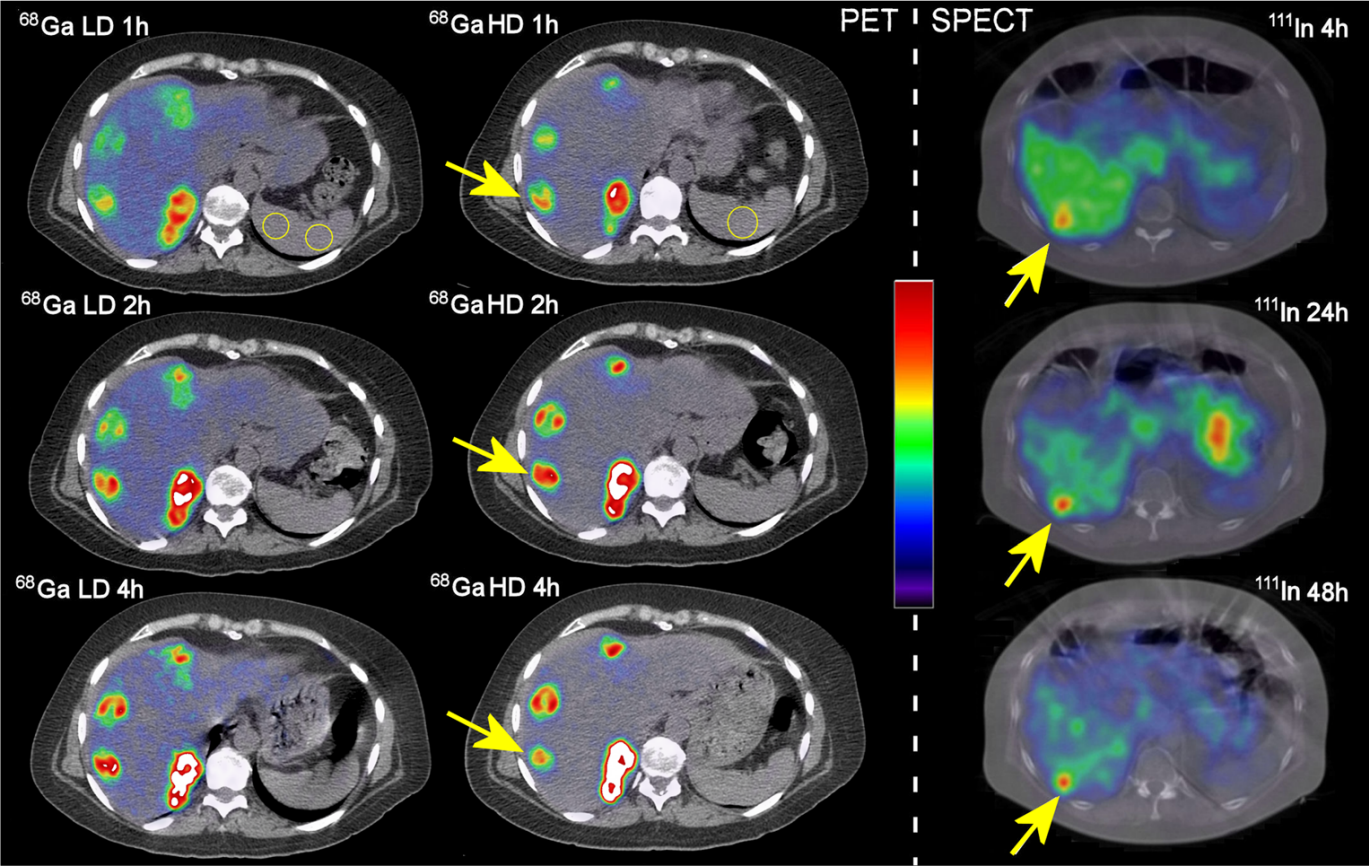
Examples of abdominal PET/CT and SPECT/CT images including normal liver, liver metastases and spleen for reference. Left column: low peptide dose [^68^Ga]-ABY-025-PET/CT at 1, 2, and 4 hours after injection. Middle column: high peptide dose [^68^Ga]-ABY-025-PET/CT at corresponding time points in the same patient. Right column (other patient): [^111^In]-ABY-025--SPECT/CT at 4, 24, and 48 hours after injection. The PET colour scale is thresholded at SUV 32. The SPECT images are corrected for the decay of ^111^In to represent uptake and the colour scale arbitrarily normalised. Arrows indicate a liver metastasis of breast cancer in each of the two patients. Examples of dual spleen VOI:s (spherical) and single slice circular ROI are shown in yellow (first row PET images). ^68^Ga LD and ^68^Ga HD stand respectively for low and high peptide content [^68^Ga]-ABY-025. ^111^In stands for [^111^In]-ABY-025

In two recent phase I/II studies, ABY-025 was successfully labelled with ^111^In for SPECT [23] and ^68^Ga for PET [24]. With both modalities, quantitative approaches were shown to distinguish HER2-negative from HER2-positive metastases. Figure 1 shows abdominal slices including spleen, liver, and liver metastases from patients with breast cancer using ^68^Ga PET and ^111^In SPECT at various time points after injection. While the data from the previous small studies are encouraging, there is a need to identify the simplest and most robust quantitative approach before the subsequent multi-centre trials in larger cohorts. The previously suggested SPECT/CT diagnostic method requires dual imaging at 4 and 24 h after injection for reliable HER2-status discrimination, which is logistically demanding in clinical routine. Ideally, SPECT/CT imaging at one time-point should be adequate. With PET we were able to show that SUV-values correlated strongly with HER2 expression based on histopathology. PET offers more accurate quantification and simpler logistics using single-time-point SUV measurements, compared to SPECT. However, use of SUV as a universal reference requires identical calibration between the scanner and well-counter equipment at various medical centres. One approach for simplified image reading and concordance among readers at different centres could be to use intra-image measurement ratios by normalising metastatic uptake to the uptake in an easily identifiable normal tissue in order to establish a single time point tumour-to-reference (T/R) ratio. This is a mainstream nuclear medicine technique for establishing diagnostic thresholds, especially with single photon methods. In addition to sub-optimal camera calibration issues, this approach also reduces the impact of many human errors such as mistyping patient weight, dose or time of injection, partly extra-vasal injection and collimator selection. The T/R concept accuracy depends on the inter-individual reference tissue uptake variation, which might vary with both imaging modality, time after injection, and amount of cold peptide co-injected with radiolabeled peptide.

In the context of imaging whole body HER2 expression there is also a need to evaluate the impact of potential binding to HER2 present in serum, as this could cause higher tracer retention in blood and tissues with high blood volume and impact the accuracy of T/R-ratios. The aims of the study presented here thus was to re-evaluate data from the two previous studies to investigate 1) the potential of a single time point T/R-ratio technique for HER2 imaging using radiolabelled ABY-025 with both SPECT and PET. 2) variation of biodistribution related to time after injection, peptide mass, and amount of shed HER2 in serum.

## Patients and methods

### Protocol outline

Image data from 94 whole-body acquisitions in 23 patients from two previous phase I studies [23, 24] were re-evaluated. Common for both studies was an initial vertex to mid-thigh [^18^F]-FDG PET/CT performed for baseline localization of viable metastases, one to seven days prior to the examination using radiolabelled ABY-025.

The first study applied SPECT/CT imaging in seven patients using [^111^In]-ABY-025 [23]: A target dose of 150 MBq [^111^In]-ABY-025 was injected and SPECT/CT of two individually selected regions of interest per patient, e.g. thorax and abdomen, were recorded at 4, 24, and 48 h after injection.

PET imaging using [^68^Ga]-ABY-025 [24]: This second study was carried out in two phases. The first phase of ten patients was performed to evaluate the impact of peptide mass and scanning time on diagnostic efficacy and involved two levels of injected ABY-025 dose (78 ± 8 μg and 427 ± 19 μg) with whole body PET/CT at 1, 2, and 4 h after injection. The second phase in six patients was a test-retest measurement designed to calculate a repeatability coefficient of lesional SUV by repeated exam with identical peptide dose and scan time, both defined after analysing the first phase results.

Detailed descriptions of radiochemistry are provided in references [23, 24]. Briefly, GMP grade ABY-025 was provided by Affibody AB in vials containing 100 μg. For SPECT 100 μg of ABY-025 was labelled with 142.6 MBq (range 131-154 MBq) of ^111^In indium. For PET, ABY-025 was labelled with 212 ± 46 MBq ^68^Ga gallium and developed separately for low (LD: 78 ± 8 μg) and high (HD: 427 ± 19 μg) ABY-025 peptide dose radiopharmaceuticals [25]. The radiochemical purity of the radiopharmaceuticals was over 95%.

Blood samples for serum-HER2 analysis (Labor Limbach, Heidelberg) were collected the day of the first radiolabelled ABY-025 injection for all patients.

### Patients

A total of 23 women with known metastatic breast cancer were included (^111^In SPECT: n = 7, ^68^Ga PET: n = 16). Potential participants were previously diagnosed with metastatic breast cancer and had known HER2 classification of the primary tumour (HER2-positive: HercepTest score 3+ or HercepTest score 2+ and ISH-positive; HER2-negative: HercepTest score 0 or 1+, or score 2+, but ISH-negative). On-going treatment, HER2-targeted or other, was not an exclusion criterion and planned medication was given as prescribed throughout the studies. Patient characteristics are summarized in Table 1. Biopsies for IHC and ISH were performed, guided by CT or ultrasound, when considered clinically relevant and tolerated by the patient. A total of 16 biopsies were obtained during the PET study and 12 during the SPECT study. IHC and ISH results were summarised as HER2-scores as described above. Details regarding handling of biopsy specimens have been published previously [23, 24].

**Table 1:** Patient characteristics for PET [24] and SPECT [23] studies

	[^68^Ga]-ABY-025-PET study			[^111^In]-ABY-025-SPECT study		
Patients	HER2-pos (n = 12)	HER2-neg (n = 4)	*P*	HER2-pos (n = 4)	HER2-neg (n = 3)	*P*
Median age in years (range)	50 (41-74)	65 (33-74)	0.43	57 (46-69)	66 (65-70)	0.21
Height (cm)	164 ± 8	165 ± 6	0.83	166 ± 5	165 ± 9	0.86
Weight (kg)	70 ± 14	64 ± 14	0.46	82 ± 3	66 ± 16	0.4
HER2-targeted treatment	8	3	0.065	4	1	0.12
[^18^F]FDG-PET/CT metastases/patient	6 ± 3	8 ± 2	0.28	13 ± 8	(65 ± 17)	0.057
Analysed biopsies	7	9	0.29	4	8	1

The original studies were academically sponsored clinical trials (trial identifiers [^111^In]-ABY-025: EudraCT 210-021078-12 and NCT01216033; [^68^Ga]-ABY-025: EudraCT 2012-005228-14 and NCT01858116), approved by the regional ethics committee and the Swedish Medical Products Agency. All patients signed informed consent.

### Image acquisition protocols

SPECT/CT (GE Infinia, GE Healthcare, Waukesha, WI, USA) was performed at 4, 24, and 48 h. At each time point two SPECT/CT scans were performed covering body parts selected from the metastatic findings in the initial [^18^F]FDG-PET/CT, typically one thoracic and one abdominal position. SPECT images were corrected for attenuation using simultaneously acquired low-dose CT, using manufacturer’s settings.

PET/CT (GE Discovery STE, GE Healthcare, Waukesha, WI, USA) was acquired by whole-body imaging from vertex to mid-thigh. All PET images underwent all relevant corrections according to the manufacturer’s recommendations. Fifteen out of 16 patients in the PET-study were scanned twice, 1 week apart. For the first ten patients, LD [^68^Ga]-ABY-025 PET preceded HD [^68^Ga]-ABY-025 PET to minimize the potential effects of prolonged peptide binding to target on the subsequent scan. These ten patients were scanned at 1 h, 2 h, and 4 h after injection to study biodistribution. Five patients were scanned twice with HD PET for test-retest purposes and were scanned only at 2 h after injection. One patient included in the test-retest experiment only underwent the first HD PET.

### Image evaluation and SUV calculations

[^18^F]FDG-PET/CT data were analysed for metastatic lesions according to routine clinical criteria by experienced observers, supported by additional imaging from various modalities when needed. Guided by findings from [^18^F]FDG-PET/CT, subsequent radiolabelled ABY-025 images were analysed for lesional uptake, which was measured as maximum voxel value. Normal tissue uptake was measured as mean of as-large-as-possible spherical VOI:s in spleen, liver, kidney, blood pool (mediastinal aorta and left ventricular cavity), muscle (erector spinae, gluteal, and shoulder), fat (subcutaneous and abdominal), and lung (dorsal, mid, and ventral). VOI values were averaged with one from each side for paired organs (three for the kidneys), three for the liver, two for spleen and aortic blood pool, and one for the cardiac blood pool. The spleen was also evaluated using a simple circular single-ROI measurement. PET measurements were converted to SUV.

For SPECT images, the attenuation corrected count values were used. When possible, the reference region was measured in the same SPECT volume because of variations in background scatter between the volumes. Scatter was generally higher in the volume closest to the kidneys. No normalization was applied when lesion and reference had to be measured in separate volumes, as this procedure would in itself also introduce a potential error source.

### Statistics

Values are reported as mean ± standard deviation (SD), unless otherwise specified. Coefficient of variation (%CV) was calculated as SD/mean. Comparisons of different settings (LD [^68^Ga]-ABY-025 versus HD [^68^Ga]-ABY-025, [^18^F]FDG vs. radiolabelled ABY-025 counterparts, HER2-positive vs. HER2-negative were assessed using the Wilcoxon rank sum test. Correlations of T/R-ratios towards SUV (PET), 24 h/4 h uptake ratios (SPECT dual time-point method) and HER2-scores were calculated using Spearman’s rank. A *P* < 0.05 was considered statistically significant.

## Results

### Protocol compliance

Twenty-two patients performed all scans according to the protocol. Patient 12 in the ^68^Ga-PET/CT study (PET#12) was recruited to the test-retest paradigm but the retest scan was cancelled because no viable metastases were detected by the initial [^18^F]FDG -PET and [^68^Ga]-ABY-025 scans. Patient clinical data is summarized in Table 1.

### Biopsies, IHC, and HER2-status in metastases

A total of 28 biopsies from 18 patients were confirmed to be metastases of breast cancer and stained for HER2-status. Remaining patients either declined biopsy or the clinical incentive was considered too weak.

### Serum-HER2 analysis

**Fig. 2:**
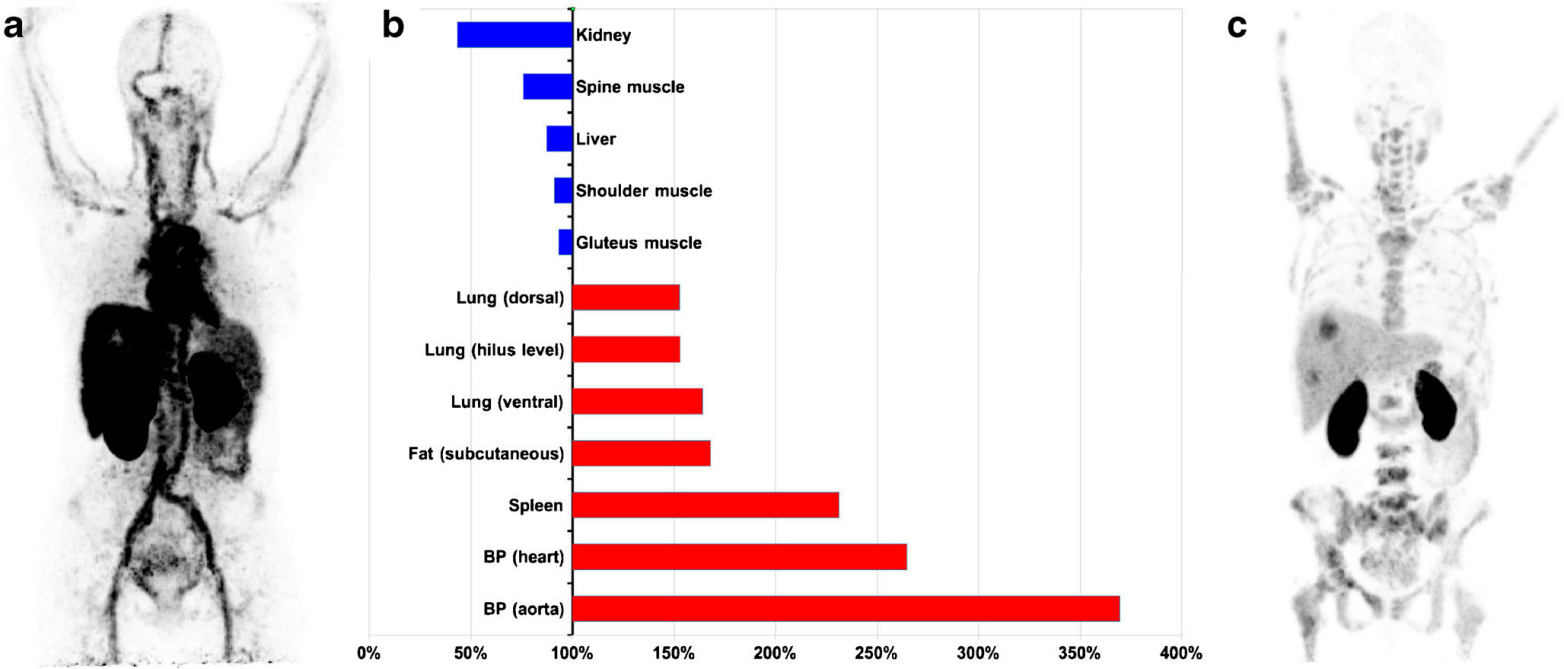
(**a**): Maximum intensity projection (MIP) example of extraordinary shedding after start of HER2-targeted therapy (PET#12) at 2 h after injection (the only available time point in this case). (**b**): Bar graph of the SUV for this patient’s tissues relative the average of all 16 patients at the corresponding time point. Highest value (370%) was found for the aortic blood pool, with 260% for cardiac blood pool (partly myocardium). Notably, a tilted distribution of activity towards blood and blood rich organs and away from kidney, muscle and liver was seen. (**c**): For comparison, a MIP of a patient with HER2-positive metastases in liver and bone marrow, but no visible activity in the blood at 2 h. This pattern was seen in 22/23 patients in both ABY-025 studies

Patient PET#12 was a clear outlier with serum-HER2 of 1276 μg/L (Normal: < 15 μg/L) [26]. The high level of shed HER2 correlated clearly with prolonged retention of radioactivity in circulation and elevated uptake in normal tissues, see Fig. 2. Excluding patient PET#12 from analyses serum-HER2 in the PET cohort was 30 ± 18 (range 11-80) μg/L and in the SPECT cohort 32 ± 17 (range 8.6-56) μg/L. Serum-HER2 did not correlate with blood pool measurements.

### Coefficient of variation

The data concerning coefficient of variation (%CV) are presented in Table 2. In both studies, the lowest tissue %CV was found for spleen (VOI) and kidney (three VOIs each), the latter with the highest accumulated tissue activity. Other examples of low %CV were cardiac blood pool and muscle followed by liver, lung, and aortic blood pool.

**Table 2:** Coefficient of variation (%CV) for tissue measurements with [^68^Ga]-ABY-025 PET in relation to injected peptide mass and time after tracer injection, and [^111^In]-ABY-025-SPECT in relation to time after tracer injection

	[^68^Ga]-ABY-025 PET						[^111^In]-ABY-025 SPECT		
	Low peptide dose			High peptide dose					
Reference tissue	1 h %CV	2 h %CV	4 h %CV	1 h %CV	2 h %CV	4 h %CV	4 h %CV	24 h %CV	48 h %CV
Spleen (VOI)	17*	18*	28**	16*	17**	19***	19*	31*	28*
Spleen (ROI)	21*	23**	27*	20**	18**	22***			
Lung (ventral)	42	39*	38***	33*	35**	35***	28	28*	31*
Lung (hilus level)	45	40	34***	28*	32**	29***	39	40*	45*
Lung (dorsal)	37	33*	31***	25*	30**	32***	32	38*	37*
Liver	30*	34*	30**	31*	33**	27***	31*	28*	27*
Kidney	20	19*	17*	16*	18*	26**	15	19*	18*
Shoulder muscle	25	31	20*	29*	19*	30*	18*	21*	24*
Dorsal spine muscle	53*	60**	35*	57*	52*	44**	10*	19*	24*
Gluteus muscle	40	38	35	35	35	35**			
Blood pool, heart	36	25*	14**	25*	18*	16*	15*	31*	19*
Blood pool, aorta	41	33	25*	37	33	28***			
Visceral fat	58*	34*	71***	53*	57	63***			
Subcutaneous fat	49	40	44*	48	48	43**	12*	22*	19*
Cerebellum	30*	30**	40**	21*	24*	43**			
Cerebrum	28	21*	25*	26*	26*	33**			

*P*-values for correlation of HER2-diagnostics with T/R and the specified tissue as reference, against biopsy analysis results for each time and dose combination coded as: * = P < 0.05, ** = P < 0.01, *** = P < 0.005

Tissues prone to metastases from breast cancer could be problematic for use as reference tissue. All visible metastases where cautiously avoided in these measurements. Bone marrow was excluded from analyses because of high degree of metastasizing in multiple patients.

### [^18^F]FDG -PET/CT

[^18^F]FDG PET/CT detected at least two lesions per patient in both studies. The difference between [^18^F]FDG SUVs for HER2-positive and negative lesions was not statistically significant either in the PET (*P* = 0.11) or SPECT study (*P* = 0.8) making tumour-to-reference calculations for HER2-expression irrelevant for [^18^F]FDG.

### Tumour-to-reference tissue (T/R) ratio

All the studied reference tissues were evaluated regarding their capacity for discriminating HER2-positive from HER2-negative metastases using the T/R approach (see Table 2). Actual reference tissue values for [^68^Ga]-ABY-025 PET are summarized in supplementary table 1. T/R-values of metastatic [^111^In]-ABY-025 SPECT voxel max to reference tissue mean were significantly different between biopsy-verified HER2-positive and negative metastases (*P* = 0.016) for all time points (4 h, 24 h, and 48 h) using spleen, muscle, or lung as reference tissue. This pattern was similar with [^68^Ga]-ABY-025 PET, with all combinations of time point and peptide dose using spleen, spine muscle, or liver as reference tissue (*P*-value range: 0.002-0.041). T/R-values for blood pool and several other tissues were likewise significant at various combinations of modality and time points. Non-significant T/R-values were most frequently seen in the earliest (1 h) PET acquisition and mainly when a low peptide amount was injected. The cardiac blood pool evaluation was the better choice with PET compared to aortic measurement (not performed for SPECT).

The best reference tissue in both modalities was spleen. T/R using spleen correlated well with IHC HER2-scores for both [^68^Ga]-ABY-025 PET (at 2 h: n = 16, r = 0.84, P < 0.001; at 4 h: n = 12, r = 0.93, P < 0.001) and [^111^In]-ABY-025 SPECT (at 24 h: n = 7, r = 0.80, P < 0.01; 48 h: n = 7, r = 0.85, P < 0.01). T/R using spleen also correlated with [^68^Ga]-ABY-025 PET SUVs (2 h: n = 16, r = 0.94, P < 0.001; at 4 h: n = 12, r = 0.96, P < 0.001).

Suitable spleen T/R cut-off levels for lesional HER2-status discrimination were identified as 2.0 for [^111^In]-ABY-025 SPECT at 24 and 48 h, and 2.75 for [^68^Ga]-ABY-025 PET at 2 h and 6.5 at 4 h. These cut-off values had accuracies >95% for discriminating IHC HER2-positive and negative metastases.

### [^68^Ga]-ABY-025 T/R reproducibility: test-retest

Five patients completed both scans in the test-retest phase of the [^68^Ga]-ABY-025 PET/CT study and were scanned using near identical radioactivity and HD peptide doses with whole-body imaging 2 h after injection one week apart. Each patient had at least two breast cancer metastases identified by [^18^F]FDG-PET and measurable in both [^68^Ga]-ABY-025 PET scans. Mean splenic SUV_mean_ with 3D-VOI was 1.7 ± 0.3 at both examinations (*P* = 0.99). T/R highly and linearly correlated with SUV_max_ for the ten metastases using both the mean of two 3D-VOI:s and a single circular 2D-ROI for defining spleen uptake (r > 0.97, *P* < 0.001 for all correlations). The repeatability coefficients of T/R (spleen) were 2.9 (15%) for the 3D-VOI and 3.3 (19%) for the 2D-ROI (*P* = 0.46). The data is presented in Table 3.

**Table 3:** Reproducibility of tumour to reference tissue ratios (T/R) for [^68^Ga]-ABY-025 PET high peptide dose at 2 hours after injection using various reference tissues

Reference tissue	Test, T/R	Retest, T/R	*P*	Repeatability coefficient (absolute)	Repeatability coefficient (relative)
Spleen (dual VOI)	8.5 ± 6.7	8.1 ± 7.0	0.74	2.90	14.6%
Spleen (simple ROI)	8.4 ± 6.6	8.6 ± 6.8	0.91	3.34	18.7%
Lung (ventral)	20.4 ± 18.6	20.1 ± 17.8	0.85	5.34	20.1%
Lung (hilus level)	20.9 ± 17.4	21.0 ± 17.0	0.91	5.01	16.4%
Lung (dorsal)	16.0 ± 12.0	15.8 ± 13.0	0.80	3.81	17.2%
Liver	3.2 ± 3.0	2.7 ± 2.1	0.91	1.72	24.0%
Kidney	0.42 ± 0.41	0.39 ± 0.36	0.74	0.222	28.2%
Blood pool (heart)	5.6 ± 4.6	5.4 ± 3.9	0.97	2.06	12.3%
Blood pool (aorta)	8.1 ± 6.0	8.8 ± 6.8	0.80	3.40	24.4%
Shoulder muscle	27.9 ± 25.3	25.0 ± 19.5	0.97	12.2	13.2%
Dorsal spine muscle	31.0 ± 22.2	29.8 ± 20.2	0.80	11.4	23.1%
Gluteal muscle	25.5 ± 22.5	25.5 ± 21.1	0.91	5.26	16.9%
Visceral fat	59.1 ± 75.3	45.3 ± 57.9	0.48	34.3	25.9%
Subcutaneous fat	74.9 ± 51.3	78.2 ± 48.8	0.97	18.6	29.7%
Cerebellum	122 ± 118	84.9 ± 66.1	0.53	135	26.9%
Cerebrum	246 ± 208	460 ± 792	0.91	1597	114%

## Discussion

**Fig. 3:**
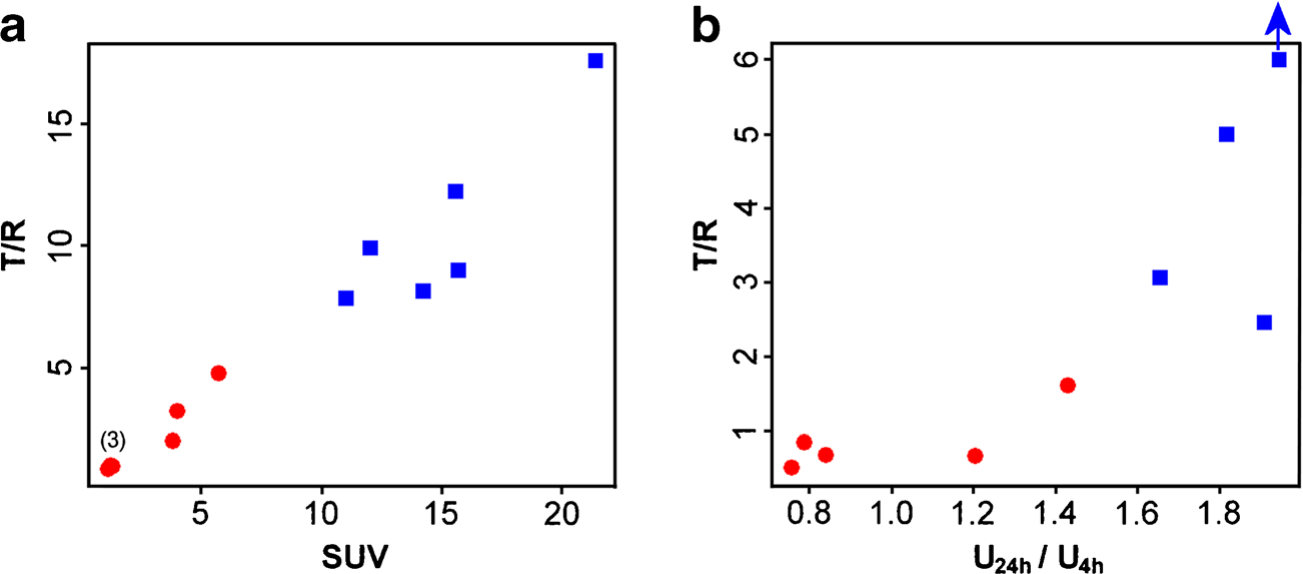
(**a**) HD [^68^Ga]-ABY-025 PET at 4 h after injection: Scatter plot of spleen T/R against SUV. Suggested cut off for T/R-based HER2-diagnostics using these criteria is 6.5. At 2 h after injection the distribution was similar, but with a suggested cut off of 2.75 with one false positive (HercepTest 2+/ISH negative) metastasis, correctly classified here at 4 h. (**b**) [^111^In]-ABY-025 SPECT at 24 h after injection. Scatter plot of spleen T/R against the previously described 24/4 h uptake ratio (dual time point) method. Suggested cutoff of 2 for HER2-diagnostics using these criteria. All data points represent metastases with HER2-status verified by immunohistochemistry. Red circles: HER2 negative, blue squares: HER2-positive. Blue arrow: actual T/R-value of 27.3

The present study investigated the feasibility of using tumour-to-reference tissue SUV ratio values obtained from whole-body HER2-imaging for standardizing the quantitative discrimination between HER2-positive and negative lesions. Data from clinical studies conducted previously in breast cancer patients using [^68^Ga]-ABY-025 PET/CT and [^111^In]-ABY-025 SPECT were used for the assessment of relevant reference tissues. For both modalities, several tissues were identified as potentially relevant. Using the spleen as reference tissue resulted in accurate discrimination of HER2-positive and negative breast cancer metastases with both modalities, see Fig. 3a and b. Using PET, the spleen T/R was associated with test-retest reproducibility similar to SUVs. This could become of value in multi-centre studies and clinical routine, if verified in larger patient cohorts.

The major criteria for the selection of a reference tissue were: 1) correlation with biopsy analysis results; 2) low variation of radioactivity uptake; 3) low probability of hosting metastases from breast cancer. Spleen was the best reference tissue for both modalities. A single circular ROI positioned centrally in the spleen was sufficient for calculating accurate and reproducible T/R-values in the current data sets. Additional tissues, such as spine muscle, lung and blood pools, could be used as alternative reference tissues, for instance in splenectomised patients. Uptake in spleen was in between the two blood pool curves and at 2 h basically the mean of these. The wash out was, however, slower for spleen than blood and even slower for lung tissue. As reference tissue, spleen, and lung could be viewed as two differently low-pass-filtered versions of the blood signal. Measurements in tissues like spleen and lung are also simpler to perform reliably than measurements in the heart, which is a moving organ, or the aorta which has somewhat limited diameter and potential variations in exact location between the CT and NM acquisition. Solid organs also generally seem less affected by shed HER2 than the blood pool itself. Undetected micrometastases present in the reference tissue might, however, result in aberrantly high values, increasing the risk of a false negative T/R value. Tissues not prone to metastases from breast cancer, therefore, appear like a safer choice, given that the other factors are equal. Potential for such micrometastases argues against liver and lung, which are otherwise convenient as reference tissue and with encouraging results for the analysed data sets.

Binding of ABY-025 to shed HER2 in blood might influence biodistribution and change the result of any method using a tissue uptake as reference. High correlation between serum-HER2 and the reference tissue uptake would indicate higher risk of diagnostic errors since high (or low) serum-HER2 cannot reliably predict the HER2-status of an individual metastatic lesion. However, serum-HER2 had minimal impact on reference tissue measurements, when the single outlier PET#12 was removed from the analysis. This patient underwent surgery for a HER2-positive breast cancer two months prior to PET. One month later she was diagnosed with massive liver metastasis and started treatment with trastuzumab, pertuzumab, and docetaxel. She was included into the test-retest arm of the PET project, where scanning with [^18^F]FDG-PET, [^68^Ga]-ABY-025 PET and diagnostic CT showed dramatic shrinkage of liver dimensions and absence of viable metastases. Serum-HER2 at the time of scanning was almost one hundred times higher than the normal upper limit. Liver biopsy after PET examination showed fibrosis and no sign of remaining cancer cells. As can be seen from Fig. 2a, [^68^Ga]-ABY-025 retention in the blood pool was very intense, resembling an angiogram. The very high serum-HER2 was associated with lower SUV in kidney, liver and muscles and higher SUV in lung, fat, spleen, and blood, which was most elevated of all tissues (Fig. 2b). The interpretation was that on-going treatment had executed a rapid cytotoxic impact on the metastases with HER2 debris leaking into the blood stream. [^68^Ga]-ABY-025 apparently identifies shed HER2 as intact HER2, resulting in increased intravascular binding in such circumstances. In the current context this patient was therefore considered an outlier. At follow-up 2 years after PET she was still in complete remission. From this single case observation it seems obvious that extremely high serum-HER2 levels can result in altered distribution of [^68^Ga]-ABY-025 amongst the normal tissues. On the other hand, serum-HER2 levels up to almost six times higher than the normal limit, as observed among the other 15 patients, did not correlate with SUV in the blood pool and did not affect the organ distribution.

The diagnostic capability of HER2 imaging using ABY-025 has previously been reported for both SPECT and PET. SUV measurements are subject to errors originating from equipment cross-calibration and data handling variation. Any selected cut-off level might carry a risk of not being fully compatible across sites and scanner models [27–29]. For multicentre studies and for daily clinical routine, a methodology independent of absolute SUV levels, based only on intra-image references, offers a simpler, more standardized and theoretically less error-prone alternative. Intra-image ratios inherently compensate for decay, administered dose and distribution volume. This was the reason why a T/R scheme was preferred over SUV for FDG PET-classification of lymphomas [29].

For SPECT such reference tissue-based methodologies are common because of even greater methodological difficulties to achieve absolute quantification. Examples for SPECT include heart-to-mediastinal ratio (H/R) in [^123^I]-MIBG neurocardiac imaging [30] and the Krenning scale (tumour-to-liver ratio) for [^111^In]-octreoscan scintigraphy [31].

If prospectively verified in subsequent clinical studies using radiolabelled ABY-025 for both PET and SPECT, a tumour-to-reference ratio using spleen, blood pool, or lung tissue might be an effective and simple approach for assessing tumour HER2 expression.

## Conclusion

Intra-image normalization of tumour-to-spleen uptake provides a simple and robust semi-quantification of HER2 expression for SPECT and PET. The diagnostic capability of spleen T/R for discriminating HER2-positive from negative breast cancer metastases was equivalent to the previously described ABY-025 methods: dual-time-point method for SPECT and SUV for PET. This simplified approach could facilitate clinical dissemination. Shedding may present diagnostic problems in the rare case with extremely high serum-HER2.

## Electronic supplementary material

Below is the link to the electronic supplementary material.

ESM 1 (DOCX 19 kb)
