# Supplementary material for: Intra-image referencing for simplified assessment of HER2-expression in breast cancer metastases using the Affibody molecule ABY-025 with PET and SPECT
Source: Eur J Nucl Med Mol Imaging. 2017 Mar 6;44(8):1337–46. doi: 10.1007/s00259-017-3650-3 (PMC5486836; doi:10.1007/s00259-017-3650-3)
Supplement: Supplementary file 1 — (DOCX 19 kb) [file 259_2017_3650_MOESM1_ESM.docx]

**Supplementary table 1. Reference tissue measurements with [^68^Ga]-ABY-025 PET of low peptide dose (LD) and high peptide dose (HD) presented as SUV ± SD.**

|  | **[^68^Ga]-ABY-025 PET** | | | | | | |
| --- | --- | --- | --- | --- | --- | --- | --- |
|  | **Low peptide dose**  **(SUV** ± SD**)** | | |  | **High peptide dose**  **(SUV** ± SD**)** | | |
| **Reference tissue** | **1h**  (n=10) | **2h**  (n=10) | **4h**  (n=10) |  | **1h**  (n=10) | **2h**  (n=16) | **4h**  (n=10) |
| **Spleen (VOI)** | 2.2 ± 0.38 | 2.1 ± 0.38 | 1.7 ± 0.48 |  | 2.0 ± 0.32 | 1.8 ± 0.61 | 1.5 ± 0.28 |
| **Lung (ventral)** | 0.52 ± 0.22 | 0.53 ± 0.21 | 0.53 ± 0.2 |  | 0.68 ± 0.22 | 0.71 ± 0.23 | 0.58 ± 0.2 |
| **Lung (hilus level)** | 0.52 ± 0.23 | 0.51 ± 0.2 | 0.52 ± 0.18 |  | 0.64 ± 0.18 | 0.67 ± 0.21 | 0.58 ± 0.17 |
| **Lung (dorsal)** | 0.62 ± 0.23 | 0.66 ± 0.22 | 0.66 ± 0.2 |  | 0.82 ± 0.2 | 0.84 ± 0.24 | 0.73 ± 0.23 |
| **Liver** | 12 ± 3.6 | 11 ± 3.6 | 8.8 ± 2.6 |  | 6.5 ± 2.0 | 5.9 ± 2.0 | 5.7 ± 1.5 |
| **Kidney** | 27 ± 5.3 | 29 ± 5.5 | 37 ± 6.5 |  | 28 ± 4.5 | 35 ± 10 | 44 ± 12 |
| **Shoulder muscle** | 0.34 ± 0.09 | 0.4 ± 0.13 | 0.39 ± 0.08 |  | 0.49 ± 0.14 | 0.51 ± 0.09 | 0.47 ± 0.14 |
| **Dorsal spine muscle** | 0.24 ± 0.13 | 0.15 ± 0.25 | 0.36 ± 0.13 |  | 0.3 ± 0.17 | 0.39 ± 0.19 | 0.33 ± 0.14 |
| **Gluteus muscle** | 0.33 ± 0.13 | 0.34 ± 0.13 | 0.43 ± 0.15 |  | 0.46 ± 0.16 | 0.49 ± 0.14 | 0.45 ± 0.16 |
| **Blood pool, heart** | 1.7 ± 0.6 | 1.6 ± 0.4 | 1.5 ± 0.21 |  | 2.5 ± 0.63 | 2.3 ± 0.85 | 1.5 ± 0.23 |
| **Blood pool, aorta** | 1.2 ± 0.5 | 1.0 ± 0.34 | 0.88 ± 0.22 |  | 1.7 ± 0.63 | 1.7 ± 0.95 | 0.93 ± 0.26 |
| **Visceral fat** | 0.39 ± 0.23 | 0.36 ± 0.12 | 0.56 ± 0.39 |  | 0.45 ± 0.24 | 0.43 ± 0.23 | 0.45 ± 0.28 |
| **Subcutaneous fat** | 0.13 ± 0.06 | 0.12 ± 0.05 | 0.14 ± 0.06 |  | 0.15 ± 0.07 | 0.16 ± 0.06 | 0.14 ± 0.06 |
| **Cerebellum** | 0.14 ± 0.04 | 0.17 ± 0.05 | 0.15 ± 0.06 |  | 0.16 ± 0.03 | 0.15 ± 0.07 | 0.14 ± 0.06 |
| **Cerebrum** | 0.07 ± 0.02 | 0.07 ± 0.01 | 0.06 ± 0.02 |  | 0.11 ± 0.03 | 0.09 ± 0.04 | 0.07 ± 0.02 |
